# Influence of PM_1_ exposure on total and cause-specific respiratory diseases: a systematic review and meta-analysis

**DOI:** 10.1007/s11356-021-16536-0

**Published:** 2021-10-09

**Authors:** Yaoyu Hu, Mengqiu Wu, Yutong Li, Xiangtong Liu

**Affiliations:** 1 Department of Epidemiology and Health Statistics, School of Public Health, Capital Medical University, No. 10 Xitoutiao, Youanmen, Fengtai District, Beijing, 100069 China; 2 Beijing Municipal Key Laboratory of Clinical Epidemiology, Beijing, 100069 China

**Keywords:** Air pollution, PM_1_, Respiratory disease, Asthma, Pneumonia, Meta-analysis

## Abstract

An increasing number of studies examined the potential effects of PM_1_ (submicronic particulate matter with an aerodynamic diameter ≤ 1 μm) on the risk of respiratory diseases; however, the results have been inconclusive. This study aimed to determine the overall association between PM_1_ with total and cause-specific respiratory diseases. A systematic review and meta-analysis was conducted with 68 related articles retrieved, and six articles met the full inclusion criteria for the final analysis. For a 10 μg/m^3^ increase in PM_1_, the pooled odds ratio (*OR*) was 1.05 (95% CI 0.98–1.12) for total respiratory diseases, 1.25 (95% CI 1.00–1.56) for asthma, and 1.07 (95% CI 1.04–1.10) for pneumonia with the *I*^2^ value of 87%, 70%, and 0%, respectively. Subgroup analyses showed that long-term exposure to PM_1_ was associated with increased risk of asthma (*OR* 1.47, 95% CI 1.33–1.63) with an *I*^2^ value of 0%, while short-term exposure to PM_1_ was not associated with asthma (*OR* 1.07, 95% CI 0.89–1.27) with the *I*^2^ value of 0%. Egger’s test showed that publication bias existed (*P* = 0.041); however, the funnel plot was symmetrical with the inclusion of the moderator. In conclusion, elevated levels of PM_1_ may increase morbidity in total and cause-specific respiratory diseases in the population.

**Supplementary Information:**

The online version contains supplementary material available at 10.1007/s11356-021-16536-0.

## Introduction

Respiratory diseases are the leading causes of morbidity and mortality worldwide (GBD 2019 Diseases and Injuries Collaborators 2020). In recent decades, chronic respiratory diseases, such as chronic obstructive pulmonary disease (COPD) and asthma, have attracted increasing attention (Wang et al. 2018; Huang et al. 2019). In 2019, the numbers of people with COPD and asthma were 212 million and 262 million worldwide, respectively (GBD 2019 Diseases and Injuries Collaborators 2020). Therefore, further study on the risk factors is necessary to minimize the morbidity of respiratory diseases and to improve prevention and guidelines for treating respiratory diseases.

Ambient particulate matter pollution has been a severe public health issue worldwide (Kim et al. 2019; Wang et al. 2021a). Although there has been improvement in air quality over recent decades in some countries, more than 90% of the global population lives in areas with air quality exceeding guidelines (Evangelopoulos et al. 2020). In 2019, ambient particulate matter pollution led about 1.4 million deaths in China (GBD 2019 Diseases and Injuries Collaborators 2020). The acute and long-term effects of ambient air pollution on human health are well known. Some epidemiological studies showed that the degree of exposure to ambient particulate matter (PM) is associated with daily mortality, mainly from cardiovascular and respiratory diseases (Liu et al. 2019a; Tian et al. 2020). Previous epidemiological studies have focused on the adverse effects of fine particulate matter (PM_2.5_, particulate matter with an aerodynamic diameter ≤ 2.5 μm) and inhalable particulate matter (PM_10_, particulate matter with an aerodynamic diameter ≤ 10 μm) (Doiron et al. 2019; Yang et al. 2020). Exposure to PM_2.5_ and PM_10_ has been proven to be associated with respiratory diseases (Yao et al. 2020; Gurung et al. 2017; Pun et al. 2017; Sicard et al. 2019; Cao et al. 2021).

The most recent research indicated that PM_1_ (submicronic particulate matter with an aerodynamic diameter ≤ 1 μm) contributed 77–86% of the PM_2.5_ concentration in China (Chen et al. 2018). However, it remains unknown whether PM_1_ or PM_1-2.5_ induced the adverse effects of PM_2.5_. It has been stated that the size of PM has a negative correlation with the level of its toxicity in the lungs, which means that PM_1_ provide more detrimental effects than PM_2.5_ (Hamra et al. 2014; Valavanidis et al. 2008). However, limited evidence was found for the association between particulate matter and respiratory diseases, especially on PM_1_.

According to previous epidemiological studies, the exposure to PM_1_ contributing to the development of respiratory diseases remains uncertain (Zhang et al. 2021). To assess the effects of exposure to PM_1_ quantitatively and accurately on the respiratory diseases, we conducted a systematic review and meta-analysis on all relevant studies published thus far.

## Methods

### Literature search strategy

This review was conducted according to the Preferred Reporting Items for Systematic Reviews and Meta-Analyses (PRISMA) statement and the Meta-analysis of Observational Studies in Epidemiology (MOOSE) guidelines (Moher et al. 2009; Stroup et al. 2000). Three authors systematically searched PubMed (1966 to Apr 2021), Embase (1950 to Apr 2021), and the Cochrane Library (2000 to Apr 2021) for studies on the associations between PM_1_ and respiratory diseases. The full search strategies are described in the supplementary data (Appendix 1). We also examined the references of the selected papers and reviews for additional pertinent data.

### Inclusion and exclusion criteria

**Fig. 1:**
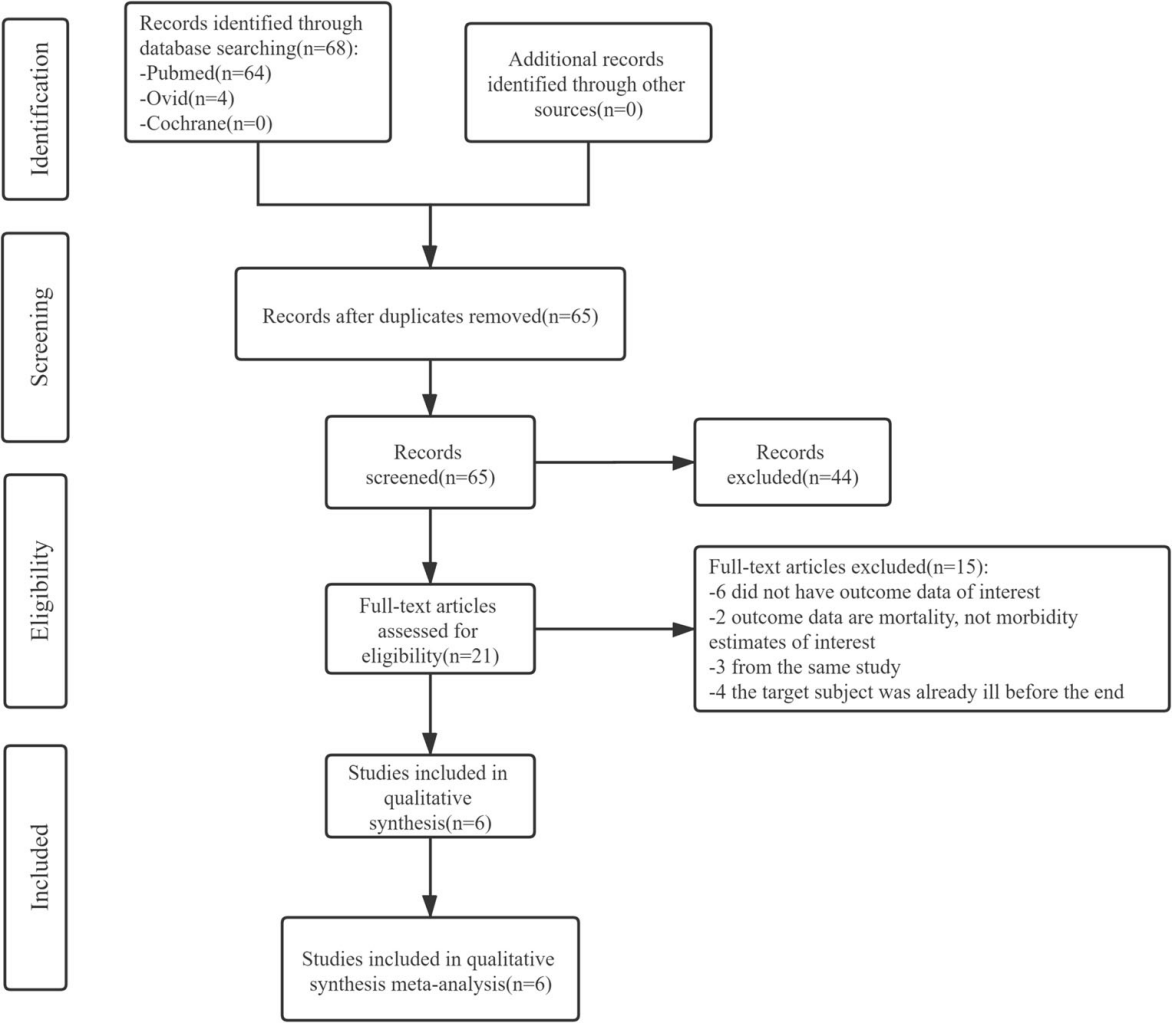
Meta-analysis flowchart of our study

The flowchart of studies through the review process is shown in Fig. 1. Studies included in this meta-analysis met the following criteria: (1) epidemiological studies investigated the association between exposure to PM_1_ and morbidity of respiratory diseases; (2) the subjects of the study were the general human population, regardless of age, geographical areas, and occupations of the population; (3) studies that quantitatively showed the results of estimation of exposure to ambient outdoor PM_1_; (4) respiratory diseases in relation to exposure to PM_1_ were selected according to the 10th Revision of the International Classification of Diseases: total respiratory diseases (ICD-10: J00-J99) and cause-specific diseases including upper respiratory tract infections (URTI, J00-J06), pneumonia (J18), obstructive pulmonary diseases (COPD, J40-J44 and J47), and asthma (J45-J46); (5) provided the effect size of prevalence, hospital admission or emergency visit of respiratory diseases per 10 or interquartile range (IQR) μg/m^3^ increase in PM_1_ concentration: regression coefficient, percentage change (*PR*), excess rate (*ER*), risk ratio (*RR*), hazard ratio (*HR*) and odds ratio (*OR*), standard error (*SE*), and/or 95% confidence interval (CI); and (6) were published in English with full text. The exclusion criteria were as follows: (1) did not conform to the inclusion criteria or (2) reviews, commentaries, or communications; or (3) the subjects of the study were patients with comorbid diseases. If studies were published based on overlapping data, the most recent article with comprehensive data was included.

### Risk of bias and quality assessment

The methodological quality and risk of bias of individual studies were assessed independently by two authors (Yaoyu Hu and Mengqiu Wu) using the criteria recommended by BioMed Central for study assessment (Luong et al. 2019). This recommendation contains 20 items for several study designs. The details of the score are as follows:

1. Overall, scores higher than 75% are considered high quality, and scores lower than 50% are not included.
2. Single questions: (1) there are only two options (yes or no): 1 is high quality, and 0 is low quality; (2) there is a problem if there are two high-quality studies and one low-quality study; (3) if there are three problems, the highest one is high quality, the middle one is medium quality, and the second one is low quality. In addition, we used RoB (Risk of Bias) for all quality evaluations.

### Data extraction

The data extraction process was independently conducted by two researchers. The extracted data included citation information (name of the first author, publication year, and location in which the study was carried out); study setting (study design, time span, sample size, age of population, and percentages of males); exposure (pollutants, exposure type, mean concentration, and method used to estimate air pollutant levels); and outcome (health outcome/diagnosis, day lags of the effect, unit of concentrations of PM_1_, confounding factors that were adjusted for and study results). The short-term effect was defined as < 7 days. For short-term exposure studies with multiple lag estimates, we used an a priori lag selection protocol devised by Atkinson et al. to select one estimate for preventing the overrepresentation of a single study in this meta-analysis (Atkinson et al. 2014).

### Statistical analysis

We used *OR* with 95% CI in the prevalence/hospital admission/emergency visit of respiratory diseases as a measure of effect size. All estimates were transformed to a 10 μg/m^3^ increase in PM_1_ concentration to pool results. We used *Q*-statistics to conduct heterogeneity tests, where *P* < 0.10 was considered to be statistically significant. The *I*^2^ statistics were calculated to represent the percentage of variation observed in studies caused by heterogeneity. An *I*^2^ value < 50% was generally regarded as low moderate heterogeneity between studies, indicating a fixed-effect model to pool the estimates. *I*^*2*^ values > 50%, representing high heterogeneity, indicated a random-effect model. We used forest plots to graphically display results. We assessed publication bias using funnel plots, contour-enhanced meta-analysis funnel plots, Begg’s test, and Egger’s weighted linear regression.

A subgroup analysis based on the exposure type of PM_1_ (short term and long term) was conducted. All tests were two-sided, and *P* < 0.05 was considered statistically significant, except in the heterogeneity test (*P* < 0.10). Transformation of effect size and meta-analysis was performed using MATLAB version 2018 and R version 4.0.5, respectively.

## Results

### Search findings and study characteristics

Sixty-eight studies were identified through the literature search, and 21 studies were eligible for full-text evaluation. Among them, 6 studies (Zhang et al. 2021; Wang et al. 2021b; Zhang et al. 2020; Yu et al. 2020; Luong et al. 2016; Michaud et al. 2004), published between 2004 and 2021, met our full inclusion criteria and were finally analyzed. The flowchart of this review shows the detailed process of selection (Fig. 1). The basic characteristics of the literature in the meta-analysis are summarized in Table 1. The included studies were performed in various regions (China, 4; Vietnam, 1; America, 1), including cross-sectional, time series, and case-crossover studies. In the included studies, PM_1_ was defined as fine particulate matter with an aerodynamic diameter ≤ 1 μm. The daily mean concentrations of PM_1_ across the 6 studies were 26.9 μg/m^3^. The outcomes of respiratory diseases included asthma (Zhang et al. 2021; Zhang et al. 2020; Yu et al. 2020; Michaud et al. 2004), pneumonia (Wang et al. 2021b; Zhang et al. 2020), and total respiratory diseases (Zhang et al. 2020; Luong et al. 2016). Four studies used ICD-10 to define the outcome, one based on incidence data, one based on emergency department visit data, and three based on hospital admission data. The estimated risk of outcome was reported as OR, HR, and PC.

**Table 1:** Summary of six articles included in the systematic review.

Study citation information and setting					Exposure			Outcome				
ID	Authors(year)	Location	Study design, Time-span	Study Size	Population (years old)	Mean/median concentration	Unit of increment (μg/m^3^)	Exposure type	Health outcome	Outcome level	Effect (95% CI)	Controlled variables
1	Wang et al. (2021b)	Hefei, China	Time series, 2016–2018	15683	0-17	31.00	10.00	Short-term, lag_0_	Pneumonia	Hospital admission, ICD-10	6.82(3.85–9.88) ^*^	Yes
2	Zhang et al. (2021)	Wuhan, China	Cross-sectional, 2014–2018	5788	3-5	37.40	10.00	Long-term	Asthma	Incidence	1.54(0.82–2.90) ^**^	Yes
3	Zhang et al. (2020)	Shenzhen, China	Case-crossover, 2015–2016	6078	All	19.00	10.00	Short-term, lag_0-2_	TRD	Hospital admission, ICD-10	1.09(1.04–1.14) ^***^	Yes
				147	All	19.00	10.00	Short-term, lag_0-2_	Asthma	Hospital admission, ICD-10	1.12(0.85–1.47) ^***^	Yes
				1661	All	19.00	10.00	Short-term, lag_0-2_	Pneumonia	Hospital admission, ICD-10	1.12(1.02–1.22) ^***^	Yes
4	Yu et al. (2020)	Liaoning, China	Cross-sectional, 2012–2013	59754	2-17	44.90	11.40	Long-term, 2009-2012	Current asthma	Prevalence	1.55(1.38–1.75) ^***^	Yes
5	Luong et al. (2016)	Hanoi, Vietnam	Case-crossover, 2010–2011	8934	0-5	54.00	42.00	Short-term, lag_0_	TRD	Hospital admission, ICD-10	1.03(1.01–1.04) ^***^	Yes
6	Michaud et al. (2004)	Hawaii, America	Time series, 1997–2001	4339	NA	1.97	10.00	Short-term, lag_0_	Asthma	Emergency department visit, ICD-9	1.03(0.90–1.42) ^****^	Yes

^*^PC; ^**^:*HR*; ^***^OR; ^****^Effect; *TRD* total respiratory diseases, *PC*percentage change, *OR* odds ratio, *HR* hazards ratio.

### Risk of bias and quality assessment

**Fig. 2:**
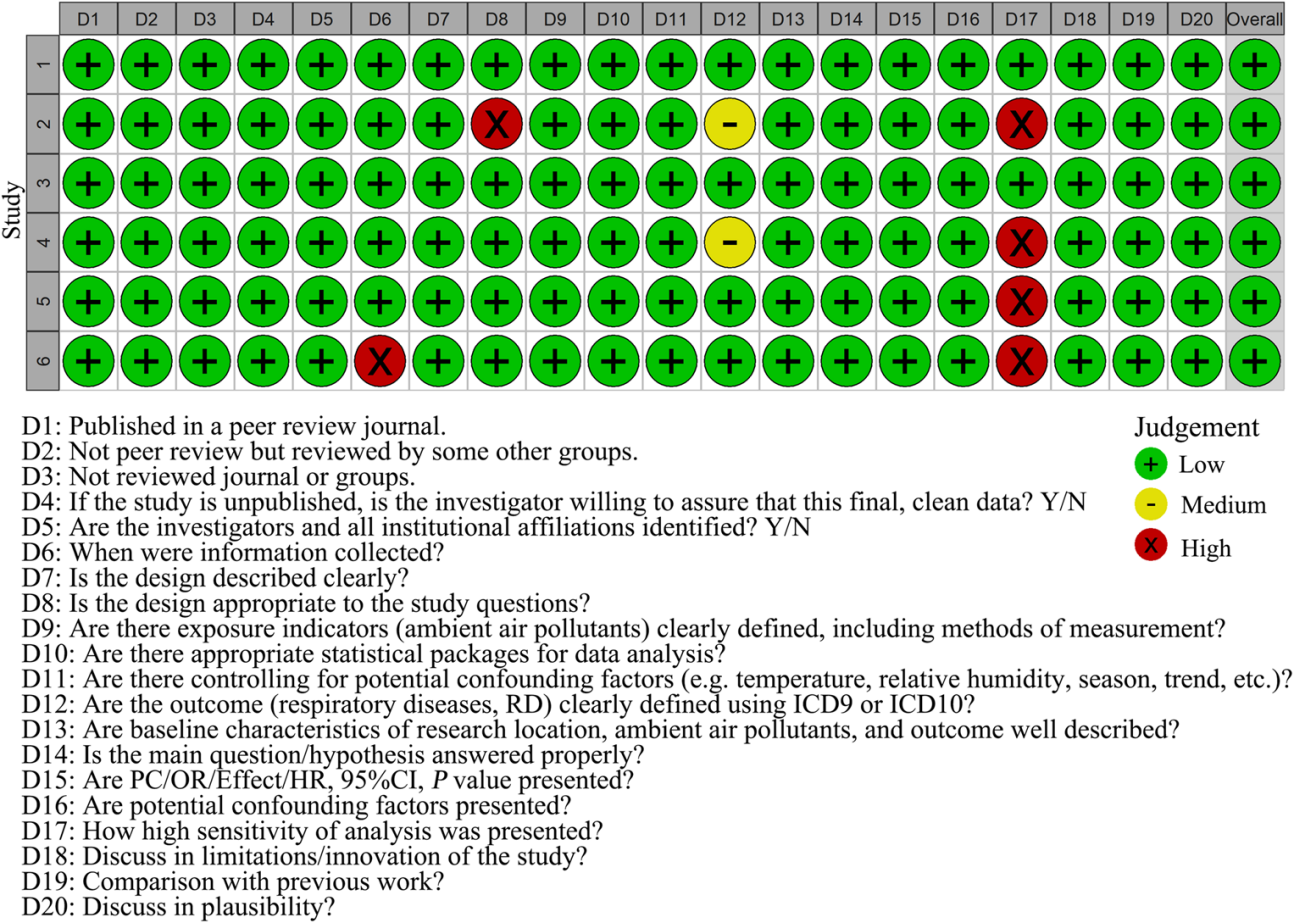
Assessment of the risk of bias in the included studies

According to our bias assessment, the six included studies all had a low risk of bias (Fig. 2, Fig. S1). Table S1 shows the detailed assessment process.

### Meta-analysis results

**Fig. 3:**
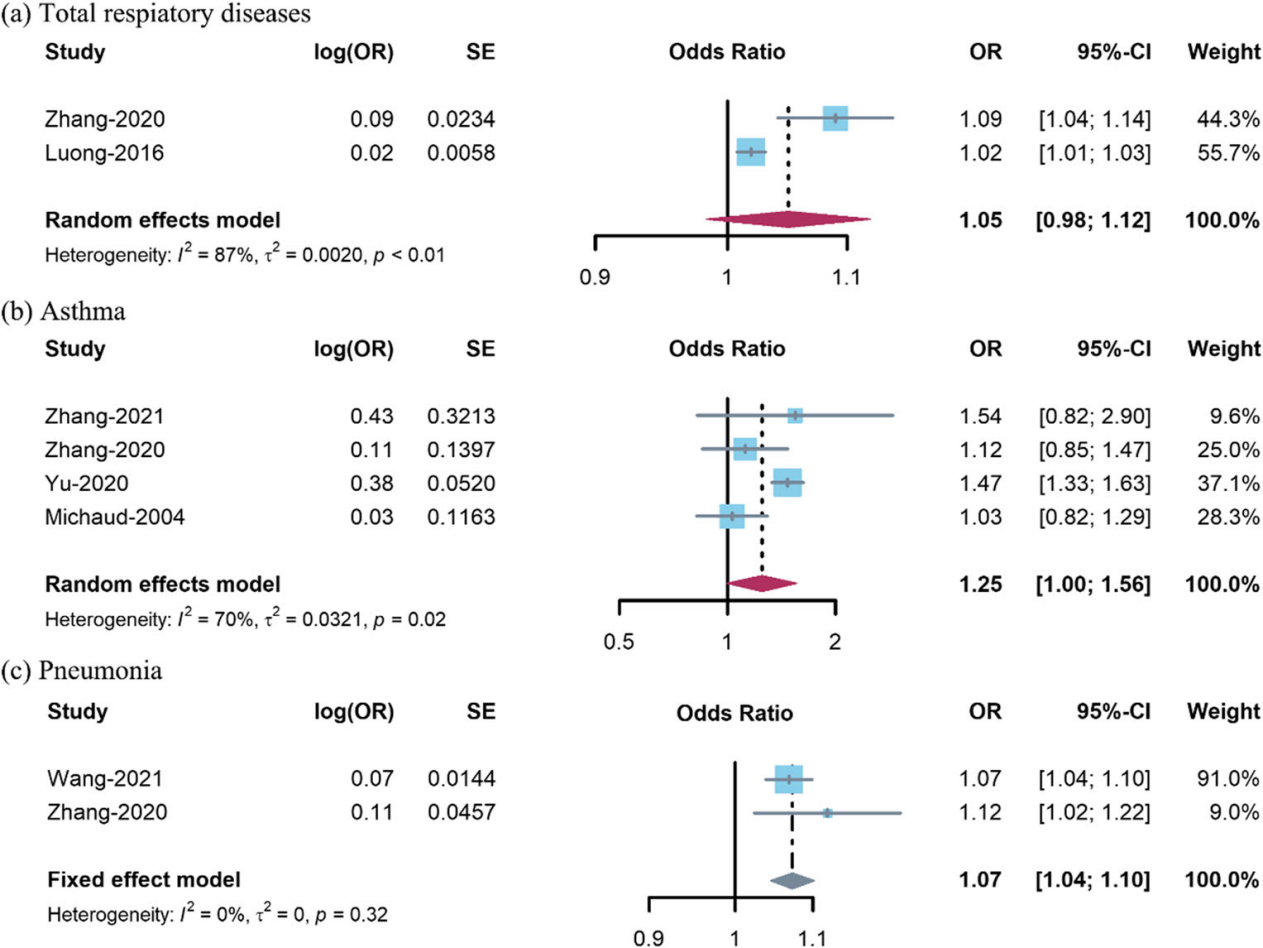
Forest plots for the association between PM_1_ and respiratory diseases

The pooled effect sizes of the association between exposure to PM_1_ and respiratory diseases are presented in Fig. 3. The degree of heterogeneity (*I*^2^) in this meta-analysis was large in pooled estimates for total respiratory diseases (87%) and asthma (70%) and low for pneumonia (0%). We revealed that the pooled effect of a 10 μg/m^3^ increase in PM_1_ on total respiratory diseases was not statistically significant (*OR* 1.05, 95% CI 0.98–1.12, Fig. 3a). There was a marginal association between a 10 μg/m^3^ increase in PM_1_ and asthma (*OR* 1.25, 95% CI 1.00–1.56, Fig. 3b). A 10 μg/m^3^ increase in PM_1_ was positively associated with pneumonia (*OR* 1.07, 95% CI 1.04–1.10, Fig. 3c).

**Fig. 4:**
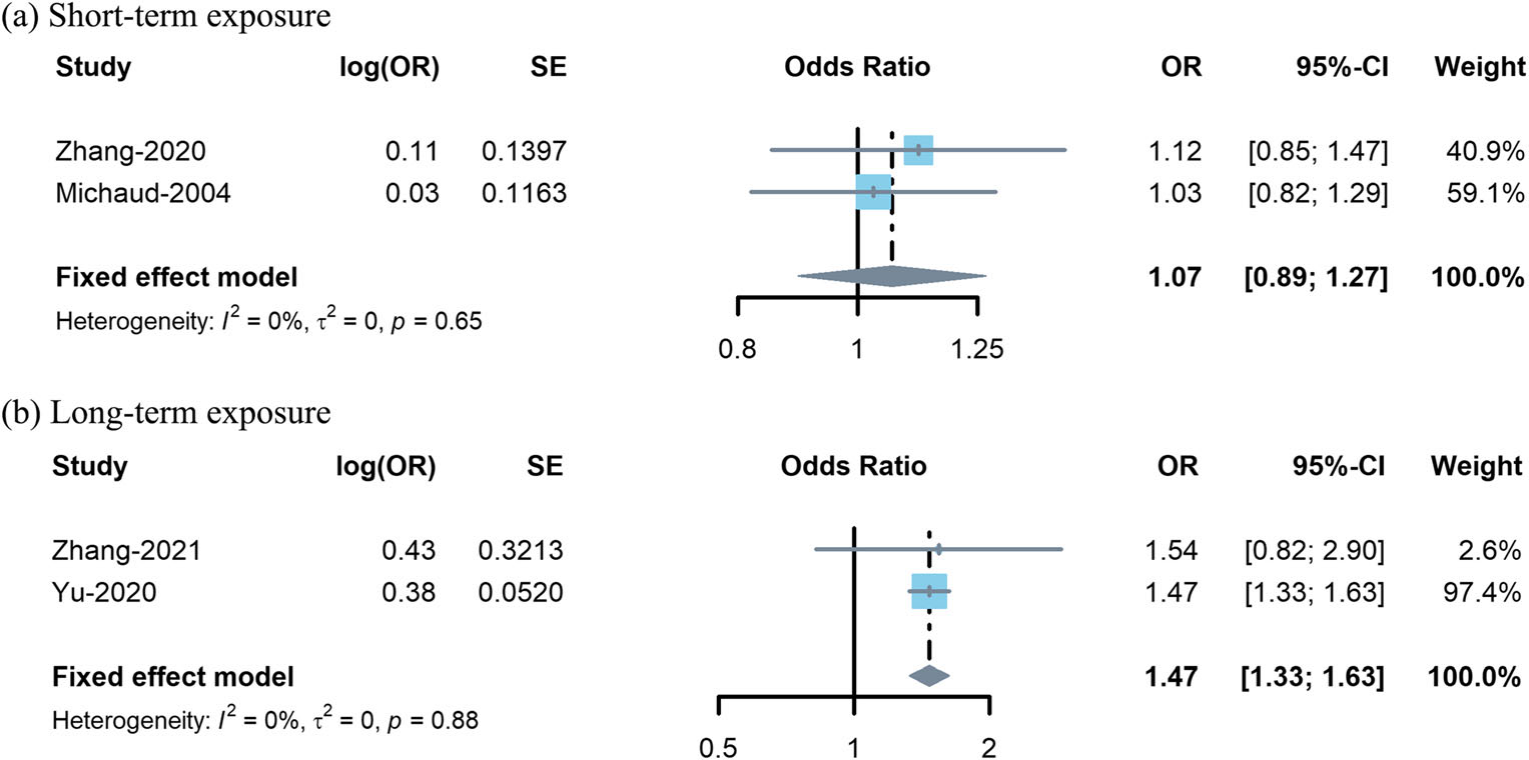
Forest plots of subgroup analysis for association between PM_1_ exposure and asthma

The subgroup analysis demonstrated that the pooled acute effect of short-term exposure to PM_1_ on asthma was not statistically significant (*OR* 1.07, 95% CI 0.89–1.27, Fig. 4a), and long-term exposure to PM_1_ was positively associated with increased risk of asthma (*OR* 1.47, 95% CI 1.33–1.63, Fig. 4b).

### Publication bias

**Fig. 5:**
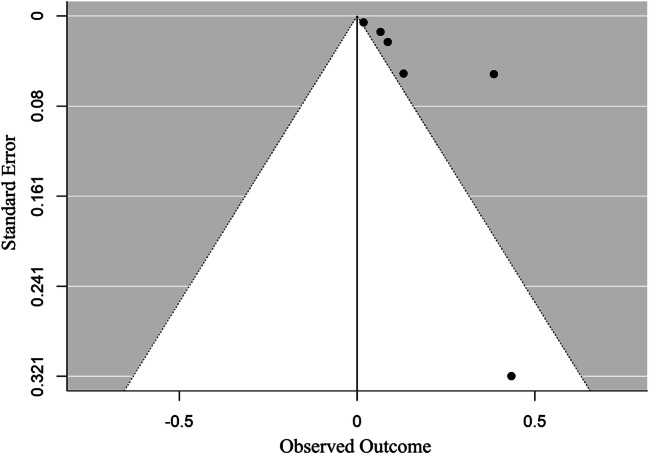
Funnel plot showing publication biases of studies on PM_1_

**Fig. 6:**
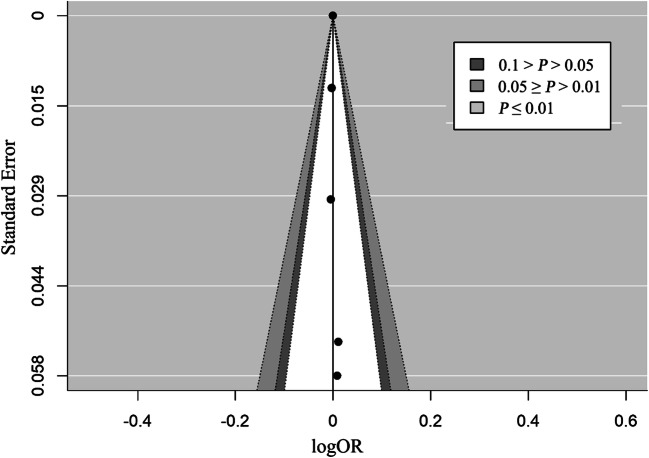
Trimmed and filled funnel plot of studies on PM_1_

The funnel plot was found to be asymmetric (Fig. 5), and publication bias was observed with Egger’s test (*P* = 0.041). Trim and fill analyses were conducted to investigate the impact of this bias (Fig. 6). With the inclusion of the moderator (study design), the funnel plot was symmetrical.

## Discussion

This is the first meta-analysis to evaluate the impact of PM_1_ on respiratory diseases and provide an estimate of the impact. We found positive associations between PM_1_ and total and cause-specific respiratory diseases. PM_1_ is a health-damaging particle because it has chemical components and can penetrate deep inside the lungs to aggravate existing asthma or contribute to chronic bronchitis development (Fuertes et al. 2014; Ostro et al. 2009). Luong et al*.* found that an increase in PM_1_ concentration would decrease pulmonary function (Luong et al. 2016). In addition, our meta-analyses showed that asthma was most strongly associated with PM_1_, which was consistent with the limited literature available on fine particles (Liu et al. 2020; Hassanvand et al. 2015). We found that the funnel graph was asymmetric, and Egger’s test observed publication bias. However, with the addition of a moderator, we found that the funnel diagram is symmetrical. This indicates that most of the heterogeneity comes from different types of research designs.

It is not easy to assess the long-term impact of PM_1_ on human health because long-term prospective observation and research require many resources and much effort. Special techniques are needed to measure and estimate the exposure level of air pollutants over a wide range of areas. The few available studies identified through our literature search reflect considerable difficulties in designing such a study to investigate the impact of PM_1_ on respiratory diseases. In addition, the results of insufficient research proved to be inconsistent. Therefore, we believe that systematic review and meta-analysis would be a good choice for a more reliable estimation of the long-term effects of PM_1_ on respiratory diseases.

Despite growing experimental evidence on the toxicity of PM_1_, whether they truly contribute to the development of clinically manifested respiratory diseases is another question. A previous analysis showed a significant correlation between PM_1_ exposure and the incidence of respiratory diseases (Yang et al. 2018). However, regarding respiratory diseases, whether PM_1_ exposure can lead to the development of the disease has been controversial. Several reports have shown a higher prevalence of acute exacerbation of respiratory diseases, and odds have reported conflicting results. Previous studies on the long-term effects of PM_1_ on respiratory diseases produced inconsistent results (Zhang et al. 2021). This inconsistency of previous studies may be based on differences in host factors that are difficult to quantify. For example, host susceptibility to air pollutants can vary widely due to genetic factors or other environmental factors, such as tobacco smoking status (Ward-Caviness 2019; Lyall et al. 2017).

The main finding of our study was a significant association between the incidence of respiratory diseases and exposure to PM_1_. However, the explanation for this result is limited because the funnel diagram is asymmetric. This is not only because of the small research influence but also because more than 10,000 participants were involved in the research, which showed relatively wide confidence intervals (Zhang et al. 2021; Wang et al. 2021b). Furthermore, the asymmetry in the funnel plot may be due to the type of study design. Although the trim-and-fill analysis showed that the pooled OR was not significantly influenced by this funnel plot asymmetry, further well-designed observational studies are still needed to better clarify the association between PM_1_ and respiratory disease development.

The issue of heterogeneity between the studies must be addressed to appreciate our results more precisely. First, these studies have different follow-up times and durations according to different study designs. Second, different studies focus on different regions, which may lead to more significant. In addition, although there is a lack of knowledge regarding how long it takes for respiratory diseases to develop from exposure to PM_1_, it is assumed that more prolonged exposure may be more harmful (Yu et al. 2020; Guan et al. 2016). This is in line with a study showing that the duration of tobacco smoking has a more substantial effect than the daily amount of cigarette consumption on the development of COPD (Bhatt et al. 2018). The lengths of the follow-up period of each study may influence the evaluation of the effect. In addition, there are differences in the methods used to estimate air pollutant levels, including ground-based monitoring stations or the space-time extremely randomized trees model. A recently adopted method uses satellite data to estimate air quality to improve the spatial and temporary resolution of air quality modeling (Wang et al. 2019). With satellite-based data, future research is expected to make it easier and more accurate to evaluate the health impact of PM_1_.

Even though the exact biological mechanism for the association between PM_1_ and respiratory diseases are not entirely clear, several studies suggested that inhalation of PM may result in inflammation and oxidative stress (Zou et al. 2020; Wang et al. 2020; Valavanidis et al. 2008). Small particles, especially PM_1_, can more easily enter and deposit in the deeper respiratory tract. After internalized by respiratory epithelial cells, PM_1_ can trigger oxidative stress and inflammatory responses (Valavanidis et al. 2008; Yang et al. 2017). Existing research indicates that the pro-inflammatory response may play important roles in the effect of PM_1_ on lung function (Mazzarella et al. 2012).

One of our study’s strengths is the efforts made throughout the design and the systematic review to ensure its validity, including the incorporating risk of bias assessment. Another strength is to search all relevant literature and to make an in-depth, transparent, and repeatable evaluation of the evidence from studies focused on PM_1_ exposures as a potential cause of respiratory diseases. It is a timely contribution to a rapidly evolving field that could inform future research's focus and design to improve its utility. However, the present study has limitations. First, the number of included studies was small, and they did not cover all countries in the world. This limitation requires further research worldwide to assess the impact of PM_1_ on respiratory diseases, which would contribute to an updated systematic review and meta-analysis. Second, most of the studies included in the meta-analysis were conducted in China, with fewer studies in high-income developed countries and low-income developing countries. Studies have shown that countries with different incomes have different air pollutant levels (Quansah et al. 2017; Liu et al. 2019b; Baumgartner et al. 2020; Naidja et al. 2018). Therefore, we need more national data to understand the health effects of PM_1_ more clearly. Finally, our meta-analysis is based on different observational research designs, including cross-sectional, case-crossover, and time series. Therefore, individual studies may be affected by uncontrolled time-varying deviations, which we could not test.

## Conclusion

The present systematic review and meta-analysis demonstrated that the pooled effect of a 10 μg/m^3^ increase in PM_1_ on total respiratory diseases was not statistically significant (*OR* 1.05, 95% CI 0.98–1.12). There was a marginal association between a 10 μg/m^3^ increase in PM_1_ and asthma (*OR* 1.25, 95% CI 1.00–1.56). A 10 μg/m^3^ increase in PM_1_ was positively associated with pneumonia (*OR* 1.07, 95% CI 1.04–1.10). Our research helps evaluate the current literature to understand the public health impact better worldwide. However, the amount of research conducted globally is still minimal, so more research needs to be carried out in different regions.

## Supplementary information

ESM 1 (DOCX 65.7 kb)

## Data Availability

Not applicable
