# Supplementary material for: Influence of PM1 exposure on total and cause-specific respiratory diseases: a systematic review and meta-analysis
Source: Environ Sci Pollut Res Int. 2021 Oct 9;29(10):15117–26. doi: 10.1007/s11356-021-16536-0 (PMC8810454; doi:10.1007/s11356-021-16536-0)
Supplement: Supplementary file 1 — (DOCX 65.7 kb) [file 11356_2021_16536_MOESM1_ESM.docx]

**Influence of PM_1_ Exposure on Total and Cause-Specific Respiratory Diseases: A Systematic Review and Meta-Analysis**

Yaoyu Hu^1, #^, Mengqiu Wu^1, #^, Yutong Li^1^, Xiangtong Liu^1, 2, *^

**^1^** School of Public Health, Capital Medical University, Beijing, 100069, China.

**^2^** Beijing Municipal Key Laboratory of Clinical Epidemiology, Beijing, 100069, China.

^#^ Yaoyu Hu and Mengqiu Wu contributed equally to this work.

**Corresponding author:**

Xiangtong Liu

Department of Epidemiology and Health Statistics

School of Public Health, Capital Medical University

No. 10 Xitoutiao, Youanmen, Fengtai District, Beijing 100069, P.R. China

Tel: (+86-010)83911778, Fax: (+86-010)83911778

E-mail: xiangtongl@ccmu.edu.cn

**Supplementary Data**

**Table of contents**

**Appendix1** Literature search strategy of our study.

**Table S1** Quality assessment of articles included in our study.

**Fig. S1** Assessment of the risk of bias in the included studies.

**Appendix 1** Literature search strategy of our study.

**Searched Database: PubMed, Embase, Cochrane library**

**1) PubMed search strategy**

**#1. COPD**

**1) PubMed search strategy**

**#1. Respiratory Tract Infections**

"Bronchitis"[Mesh] OR "Respiratory Tract Infection" [Mesh] OR "Common Cold"[Mesh] OR "Influenza, Human"[Mesh] OR " Rhinitis " [Mesh] OR " Tracheitis " [Mesh] OR " Pharyngitis "[Mesh] OR "Laryngitis"[Mesh] OR "Respiratory Tract Infection" [tiab]

**#2. Asthma**

“asthma”[Mesh] OR “Asthma, Aspirin-Induced”[Mesh] OR “Asthma, Exercise-Induced”[Mesh] OR “Asthma, Occupational”[Mesh] OR “Status Asthmaticus”[Mesh] OR “Asthma, Bronchial”[Mesh] OR “asthma”[tiab] OR “bronchial asthma”[tiab] OR “bronchial disorder”[tiab] OR “airway inflammation”[tiab] OR “wheeze”[tiab] OR “wheezing”[tiab] OR “allergy”[tiab] OR “allergic disorder”[tiab]

**#3. COPD**

"Lung Diseases, Obstructive"[Mesh:noexp] OR "Pulmonary Disease, Chronic Obstructive"[Mesh] OR "Pulmonary Emphysema"[Mesh] OR “Bronchitis, chronic”[Mesh] OR **“**Chronic obstructive pulmonary disease"[tiab] OR "Emphysema"[tiab] OR "Chronic bronchitis"[tiab] OR "Chronic obstructive lung disease"[tiab] OR "COPD"[tiab]

**#4. Pneumonias**

"Pneumonia"[Mesh] OR "Bronchopneumonia"[Mesh] OR "Pneumonia, Bacterial"[Mesh] OR "Pneumonia, Necrotizing"[Mesh] OR "Pneumonia, Viral"[Mesh] OR "Pneumonia"[tiab]

**#5. Human Influenzas**

" Human Influenzas "[Mesh] OR “Influenza”[Mesh] OR “Human Flu”[Mesh]

**#6. PM_1_**

"PM1"[tiab] OR "PM(1)"[tiab] OR "sub-micrometric Particulate Matter"[tiab]

**# Final search strategy: (#1 OR #2 OR #3 OR #4 OR #5) AND #6**

**2) Embase search strategy**

**#1. Respiratory Tract Infections**

'Bronchitis'/exp OR 'Respiratory Tract Infection'/exp OR 'Common Cold'/exp OR 'Influenza, Human'/exp OR 'Rhinitis'/exp OR 'Tracheitis'/exp OR 'Pharyngitis'/exp OR 'Laryngitis'/exp OR 'Respiratory Tract Infection':ab,ti

**#2. Asthma**

'asthma'/exp OR 'Asthma, Aspirin-Induced'/exp OR 'Asthma, Exercise-Induced'/exp OR 'Asthma, Occupational'/exp OR 'Status Asthmaticus'/exp OR 'Asthma, Bronchial'/exp OR 'asthma'/exp OR 'bronchial asthma'/exp OR 'bronchial disorder'/exp OR 'airway inflammation':ab,ti OR 'wheeze':ab,ti OR 'wheezing':ab,ti OR 'allergy':ab,ti OR 'allergic disorder':ab,ti

**#3. COPD**

'Chronic obstructive lung disease'/exp OR 'Chronic bronchitis'/exp OR 'Lung emphysema'/exp OR 'Chronic obstructive pulmonary disease':ab,ti OR 'Emphysema':ab,ti OR 'Chronic bronchitis':ab,ti OR 'Chronic obstructive lung disease':ab,ti OR 'COPD':ab,ti

**#4. Pneumonias**

'Pneumonia'/exp OR 'Bronchopneumonia'/exp OR 'Pneumonia, Bacterial'/exp OR 'Pneumonia, Necrotizing'/exp OR 'Pneumonia, Viral'/exp OR 'Pneumonia':ab,ti

**#5. Human Influenzas**

'Human Influenzas '/exp OR “Influenza'/exp OR “Human Flu'/exp

**#6. PM_1_**

'PM1/exp OR PM(1):ab,ti OR 'sub-micrometric Particulate Matter:ab,ti

**# Final search strategy: (#1 OR #2 OR #3 OR #4 OR #5) AND #6**

**3) Cochrane Library search strategy**

**#1. Respiratory Tract Infections**

MeSH descriptor: [Influenza, Human] explode all trees

OR

Bronchitis:ti,ab,kw OR Respiratory Tract Infection:ti,ab,kw OR Common Cold:ti,ab,kw OR Influenza, Human:ti,ab,kw OR Rhinitis:ti,ab,kw OR Tracheitis:ti,ab,kw OR Pharyngitis:ti,ab,kw OR Laryngitis:ti,ab,kw OR Respiratory Tract Infection:ti,ab,kw

**#2. Asthma**

MeSH descriptor: [Asthma] explode all trees

OR

asthma:ti,ab,kw OR bronchial asthma:ti,ab,kw OR bronchial disorder:ti,ab,kw OR airway inflammation:ti,ab,kw OR wheeze:ti,ab,kw OR wheezing:ti,ab,kw OR allergy:ti,ab,kw OR allergic disorder:ti,ab,kw

**#3. COPD**

MeSH descriptor: [Pulmonary Disease, Chronic Obstructive] explode all trees

OR

Chronic obstructive pulmonary disease:ti,ab,kw OR Emphysema:ti,ab,kw OR Chronic bronchitis:ti,ab,kw OR Chronic obstructive lung disease:ti,ab,kw OR COPD:ti,ab,kw

**#4. Pneumonias**

MeSH descriptor: [Pneumonia, Necrotizing] explode all trees

OR

Pneumonia:ti,ab,kw OR Bronchopneumonia:ti,ab,kw OR Pneumonia, Viral:ti,ab,kw OR Pneumonia:ti,ab,kw

**#5. Human Influenzas**

MeSH descriptor: [Human Influenzas] explode all trees

OR

Influenza:ti,ab,kw OR Human Flu:ti,ab,kw

**#6. PM_1_**

PM1:ti,ab,kw OR PM(1):ti,ab,kw OR sub-micrometric Particulate Matter:ti,ab,kw

**# Final search strategy : (#1 OR #2 OR #3 OR #4 OR #5) AND #6**

**Table S1** Quality assessment of articles included in our study.

| Criteria |  | Question | Score | Max Score | Wang et al. | Zhang et al. | Zhang et al. | Yu et al. | Luong et al. | Michaud et al. |
| --- | --- | --- | --- | --- | --- | --- | --- | --- | --- | --- |
|  |  |  |  |  | 2021 | 2021 | 2020 | 2020 | 2016 | 2004 |
| Source of the Information | 1 | Published in a peer review journal | 3 | 3 | 3 | 3 | 3 | 3 | 3 | 3 |
|  | 2 | Not peer review but reviewed by some other groups | 2 |  | 0 | 0 | 0 | 0 | 0 | 0 |
|  | 3 | Not reviewed journal or groups | 1 |  | 0 | 0 | 0 | 0 | 0 | 0 |
|  | 4 | Unpublished |  |  |  |  |  |  |  |  |
|  |  | If the study is unpublished, is the investigator willing to assure that this final, clean data? Y/N | 0 | 0 | 0 | 0 | 0 | 0 | 0 | 0 |
|  | 5 | Are the investigators and all institutional affiliations identified? Y/N | 1 | 1 | 1 | 1 | 1 | 1 | 1 | 1 |
|  | 6 | When were information collected |  |  |  |  |  |  |  |  |
|  |  | Recent 5 years | 3 | 3 | 3 | 3 | 3 | 3 | 3 | 0 |
|  |  | Recent 10 years | 2 |  | 0 | 0 | 0 | 0 | 0 | 0 |
|  |  | More than 10 years | 1 |  | 0 | 0 | 0 | 0 | 0 | 1 |
| Study Design | 7 | Is the design described clearly? | 1 | 1 | 1 | 1 | 1 | 1 | 1 | 1 |
|  | 8 | Is the design appropriate to the study questions? |  | 4 |  |  |  |  |  |  |
|  |  | Case reports and case series | 0 |  |  |  |  |  |  |  |
|  |  | Cross-sectional studies | 1 |  | 1 | 1 |  | 1 |  | 1 |
|  |  | Case-control studies | 2 |  |  |  |  |  |  |  |
|  |  | Cohort /Panel studies | 3 |  |  |  |  |  |  |  |
|  |  | Time series / Case-crossover studies | 4 |  |  |  | 4 |  | 4 |  |
|  | 9 | Are there exposure indicators (ambient air pollutants) clearly defined, including methods of measurement? |  |  |  |  |  |  |  |  |
|  |  | Clearly definition | 1 | 1 | 1 | 1 | 1 | 1 | 1 | 1 |
|  |  | Change in one unit of air pollutants | 1 | 1 | 1 | 1 | 1 | 1 | 1 | 1 |
|  | 10 | Are there appropriate statistical packages for data analysis? | 1 | 1 | 1 | 1 | 1 | 1 | 1 | 1 |
|  | 11 | Are there controlling for potential confounding factors (e.g. temperature, relative humidity, season, trend, DOW, etc.)? | 1 | 1 | 1 | 1 | 1 | 1 | 0 | 0 |
|  | 12 | Are the outcome (wheezing symptom diseases) clearly defined using ICD9 or ICD10? | 2 | 2 | 2 | 0 | 2 | 0 | 2 | 0 |
|  |  | General group of wheeze associated disorder | 1 | 1 | 1 | 1 | 1 | 1 | 1 | 1 |
|  |  | Cause-specific Wheeze associated disorder | 1 | 1 | 1 | 1 | 1 | 1 | 0 | 1 |
| Study Results | 13 | Are baseline characteristics of research location, ambient air pollutants, and outcome well described? | 1 | 1 | 1 | 1 | 1 | 1 | 1 | 1 |
|  | 14 | Is the main question/hypothesis answered properly? | 1 | 1 | 1 | 1 | 1 | 1 | 1 | 1 |
|  | 15 | Are PC/OR/Effect/HR, 95%CI, *P* value presented? | 2 | 2 | 2 | 2 | 2 | 2 | 2 | 2 |
|  | 16 | Are potential confounding factors presented? | 1 | 1 | 1 | 1 | 1 | 1 | 1 | 1 |
|  | 17 | How high sensitivity of analysis was presented? |  |  |  |  |  |  |  |  |
|  |  | Degrees of freedom (for season and trend) | 1 | 1 | 1 | 1 | 1 | 0 | 1 | 0 |
|  |  | Other factor classification (gender, etc.) | 1 | 1 | 1 | 1 | 0 | 1 | 0 | 1 |
| Study Discussion | 18 | Discuss in limitations/innovation of the study? | 1 | 1 | 1 | 1 | 1 | 1 | 1 | 1 |
|  | 19 | Comparison with previous work? | 1 | 1 | 1 | 1 | 1 | 1 | 1 | 1 |
|  | 20 | Discuss in plausibility? | 1 | 1 | 1 | 1 | 1 | 1 | 1 | 1 |
| Total score | | |  | 30 | 27 | 25 | 29 | 24 | 27 | 21 |


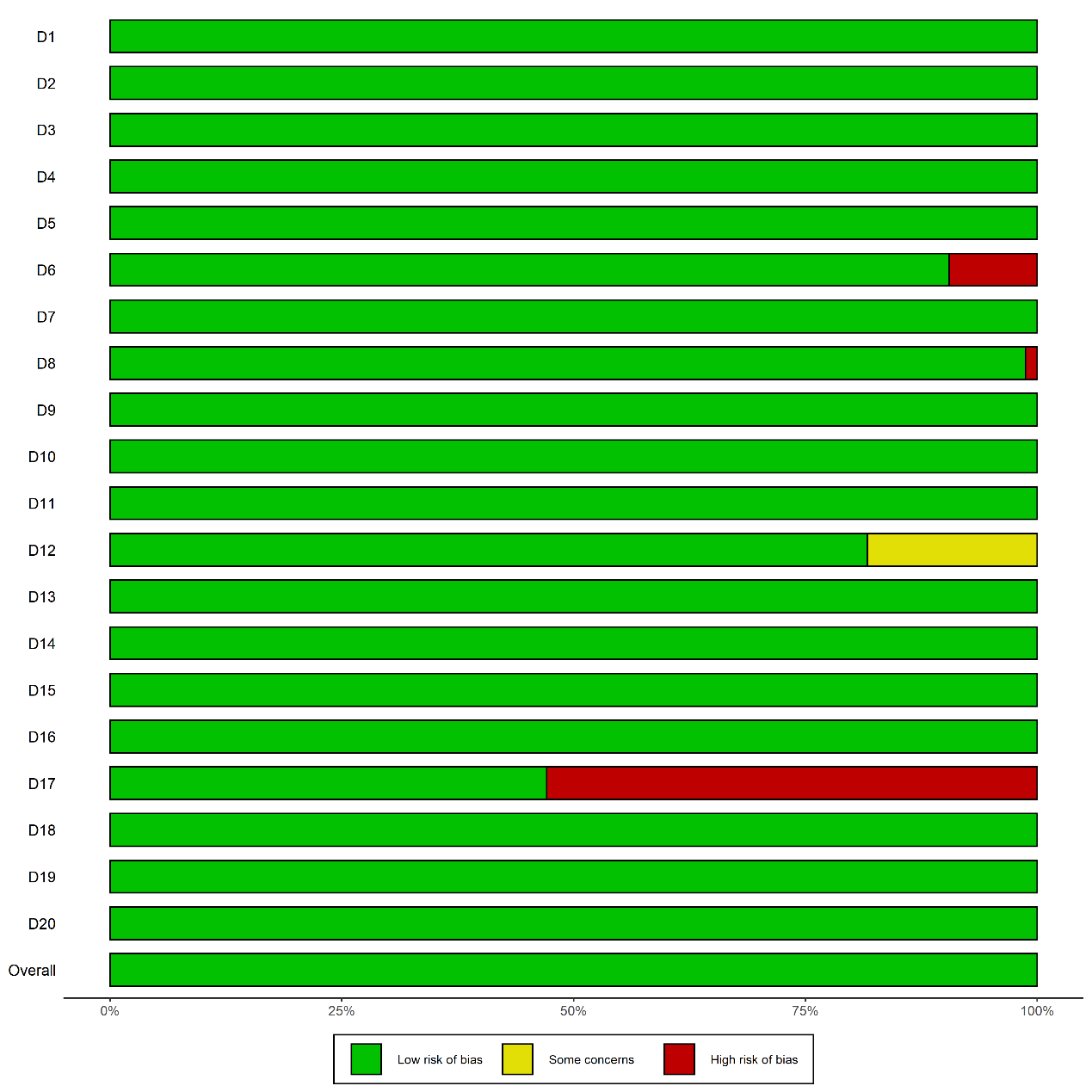


**Fig. S1.** Assessment of the risk of bias in the included studies.
